# Advances in Research on the Regulation of Floral Development by *CYC*-like Genes

**DOI:** 10.3390/cimb45030131

**Published:** 2023-03-02

**Authors:** Yuhong Chai, Hua Liu, Wendan Chen, Chenghu Guo, Haixia Chen, Xi Cheng, Dongliang Chen, Chang Luo, Xiumei Zhou, Conglin Huang

**Affiliations:** 1 School of Horticulture and Landscape Architecture, Henan Institute of Science and Technology, Xinxiang 453003, China; 2 Institute of Grassland, Flowers and Ecology, Beijing Academy of Agriculture and Forestry Sciences, Beijing 100097, China; 3 Engineering Technology Research Center of Characteristic Horticultural Plant Development and Utilization, Henan Institute of Science and Technology, Xinxiang 453003, China

**Keywords:** *CYCLOIDEA* (*CYC*)-like gene, TCP gene family, CYC2 clade, floral symmetry, molecular regulatory mechanism, phylogeny

## Abstract

*CYCLOIDEA* (*CYC*)-like genes belong to the TCP transcription factor family and play important roles associated with flower development. The *CYC*-like genes in the CYC1, CYC2, and CYC3 clades resulted from gene duplication events. The CYC2 clade includes the largest number of members that are crucial regulators of floral symmetry. To date, studies on *CYC*-like genes have mainly focused on plants with actinomorphic and zygomorphic flowers, including *Fabaceae*, *Asteraceae*, *Scrophulariaceae*, and *Gesneriaceae* species and the effects of *CYC*-like gene duplication events and diverse spatiotemporal expression patterns on flower development. The *CYC*-like genes generally affect petal morphological characteristics and stamen development, as well as stem and leaf growth, flower differentiation and development, and branching in most angiosperms. As the relevant research scope has expanded, studies have increasingly focused on the molecular mechanisms regulating *CYC*-like genes with different functions related to flower development and the phylogenetic relationships among these genes. We summarize the status of research on the *CYC*-like genes in angiosperms, such as the limited research conducted on CYC1 and CYC3 clade members, the necessity to functionally characterize the *CYC*-like genes in more plant groups, the need for investigation of the regulatory elements upstream of *CYC*-like genes, and exploration of the phylogenetic relationships and expression of *CYC*-like genes with new techniques and methods. This review provides theoretical guidance and ideas for future research on *CYC*-like genes.

## 1. Introduction

Cubas et al. first proposed the concept of the TCP transcription factor family, which is named according to the first letters of TEOSINTE BRANCHED 1 (TB1) in maize (*Zea mays*), CYCLOIDEA (CYC) in snapdragon (*Antirrhinum majus*), and PROLIFERATING CELL FACTOR 1 and 2 (PCF1 and PCF2) in rice (*Oryza sativa*) [1,2,3,4,5]. Genes encoding proteins with the TCP domain are involved in the regulation of angiosperm growth and development [6,7,8,9]. The TCP family members contain a highly conserved TCP domain, which forms a basic helix–loop–helix (bHLH) structure associated with DNA binding and protein dimerization [10,11]. TB1 is a major regulator of stem and lateral bud growth and the male flower formation of maize, rice, wheat, and other crops [12,13,14], whereas CYC controls the floral dorsal organ characteristics in snapdragon [1,15], and both PCF1 and PCF2 bind to the promoter of PROLIFERATING CELL NUCLEAR ANTIGEN (PCNA), which is crucial for DNA replication and repair, chromatin structure maintenance, chromosome isolation, and the cell cycle in rice [3]. According to their different domains, the members of the TCP family have been divided into the following two categories: TCP-P and TCP-C [16,17,18]. Moreover, TCP-C has been subdivided into the ECE (CYC/TB1) and CINCINNATA (CIN) clades [19,20].

**Figure 1:**
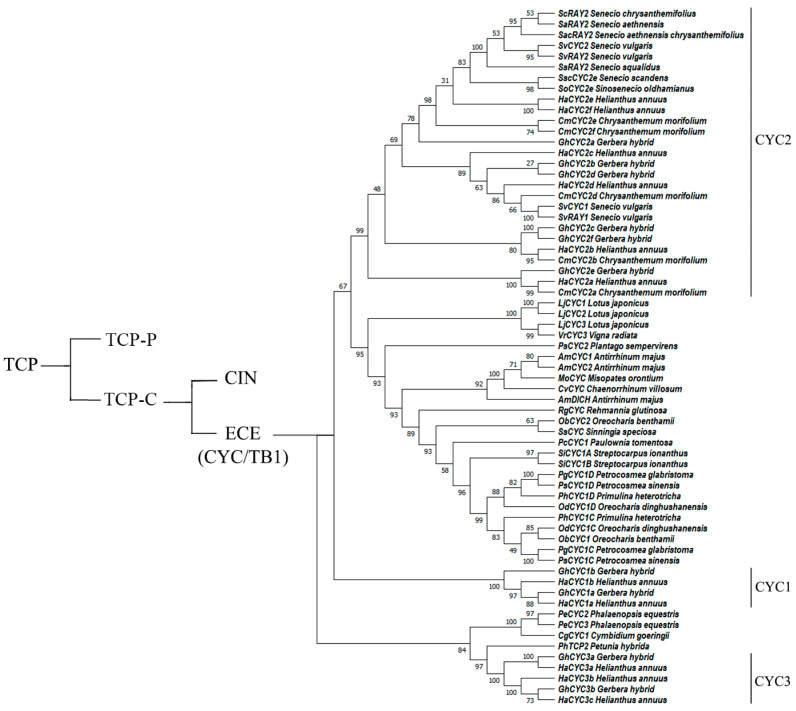
Phylogenetic tree of selected *CYC*-like genes in angiosperms. The number beside each node is the bootstrap support value.

The *CYC* genes belong to the ECE clade, which is unique to angiosperms [21,22]. In addition to the TCP and R domain sequences, *CYC* genes encode the glutamate–cysteine–glutamic acid (ECE) motif specific to core eudicots [23,24,25]. Phylogenetic analysis has indicated that *CYC* genes in angiosperms experienced two major gene duplication events, which led to the formation of the CYC1, CYC2, and CYC3 clades [26,27,28]. In different evolutionary lineages, gene duplication events occurred in each branch at different time points during evolution [29,30,31,32,33,34,35], as shown in Figure 1. A more thorough analysis of the CYC2 subgroup confirmed that they are key regulatory genes for the bilateral symmetry of flowers [36,37,38].

Angiosperm flowers can be divided into the following three types according to the presence or absence of a plane of symmetry: (1) radially symmetrical flowers (actinomorphic) with multiple planes of symmetry, (2) bilaterally symmetrical flowers (zygomorphic) with only one plane of symmetry, and (3) asymmetrical flowers with no plane of symmetry [39,40,41,42]. Studies of fossils have revealed that angiosperm flowers were originally actinomorphic and that zygomorphic flowers arose during evolution [43,44,45,46]. Wild-type snapdragon, which has typical zygomorphic flowers, is a model plant for studying floral symmetry [47]. Early investigations on the molecular mechanism regulating snapdragon flower types demonstrated that zygomorphy was determined by *CYC*, *DICHOTOMA* (*DICH*), *RADIALIS* (*RAD*), and *DIVARICATA* (*DIV*) genes encoding regulators of the dorsoventral characteristics of flowers [1,4]. The functionally redundant *CYC* and *DICH* genes belong to the CYC2 clade and originated from gene duplication events associated with angiosperm evolution [48,49,50]. These genes are essential for the development of zygomorphic flowers in angiosperms, especially the *CYC* genes [45,51,52]. The results of studies on snapdragon compelled researchers to investigate the mechanism by which *CYC* genes regulate floral symmetry. In addition to snapdragon, the regulatory function of *CYC* genes has been explored in other insect-pollinated plants with zygomorphic flowers, including *Fabaceae*, *Gesneriaceae*, *Caprifoliaceae*, *Scrophulariaceae*, and *Malpighiaceae* species [53,54,55,56,57]. The results of these studies suggest that *CYC2* genes are the key genes for regulating the development of zygomorphic flowers.

This review summarizes the status of research and unresolved problems in *Fabaceae*, *Asteraceae*, *Scrophulariaceae*, *Gesneriaceae*, *Orchidaceae*, *Solanaceae*, and other families, while also proposing future directions for studies on *CYC*-like genes. As the three largest families of angiosperms, *Fabaceae*, *Asteraceae*, and *Orchidaceae* have been the main focus of research on *CYC*-like genes. In the *Fabaceae* species, *CYC*-like genes control floral symmetry, whereas the *CYC*-like genes in the *Asteraceae* species modulate changes to the capitulum morphology and regulate elongation of the showy corolla limb of the ray florets. In the *Scrophulariaceae* and *Gesneriaceae* species, *CYC*-like genes mainly affect the morphological characteristics of petals and stamen development. The *CYC*-like genes in the *Orchidaceae* species influence floral development or branching. The *CYC*-like genes in the *Solanaceae* species mainly affect axillary bud growth and development and stem growth, while also controlling branching, flower differentiation and development, and leaf size. These genes contribute to morphological changes to the dorsoventral floral symmetry in the *Caprifoliaceae* species. A systematic analysis of the functions and evolution of *CYC*-like genes may provide researchers and breeders with a theoretical basis for future research.

## 2. Progress in Research on *CYC*-like Genes in *Fabaceae*

The *Fabaceae* species are distributed worldwide. Because of their diversity in floral symmetry, legumes are suitable for exploring the evolution and underlying mechanism of floral symmetry [58]. Researchers have screened the *Fabaceae* for homologs of snapdragon *CYC* genes and then analyzed their functions to clarify the role of *CYC*-like genes in angiosperm floral development. The differences among the diverse species in terms of the *CYC*-like genes responsible for floral symmetry revealed a new regulatory system.

The duplication of *CYC* homologues gave rise to three copies of ECE clade genes in the TCP family in *Lotus Japonicus* [59]. In *L. japonicus*, both *LjCYC1* and *LjCYC2* mediate the development of asymmetrical inflorescences and flowers, and changes in the number of petals and in wing and keel morphology were observed in transgenic plants separately overexpressing *LjCYC1* and *LjCYC2* [59]. The asymmetrical expression pattern of *LjCYC2* is similar to that of the snapdragon *CYC* gene in the developing flower primordium [59]. However, *LjCYC2* is also expressed in the inflorescence primordium of *L. japonicus*, whereas the *CYC* gene is expressed only during floral primordium development in snapdragon [4].

**Figure 2:**
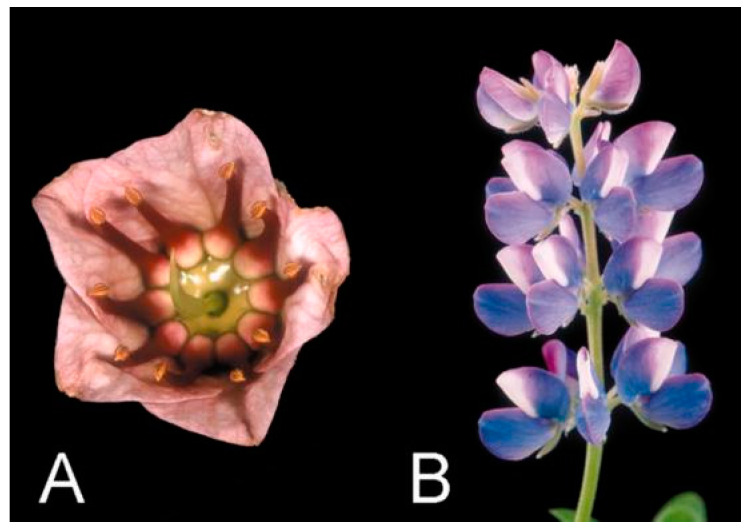
Flowers of *Cadia purpurea* and *Lupinus nanus* [60]. (**A**) Flower of *Cadia purpurea* with actinomorphic corolla. (**B**) Flowers of *Lupinus nanus* with zygomorphic corolla. Reprinted with permission from Ref. [60]. Copyright © 2023, Oxford University Press.

Citerne et al. reported that the homologous genes of *CYC* in legumes can be divided into two major classes, ECE groups I and II, which are the result of an early duplication event [60]. ECE I can be further divided into two subclasses, IA and IB, which originated from duplication near or prior to the divergence of legumes. The *LEGCYC* genes in *Lupinus* are homologous to the regulatory gene *CYC* that controls the floral symmetry and paraxial floral organ characteristics of snapdragon and its related species [61]. Ree et al. suggested based on a molecular evolutionary analysis that positive selection has played a role in the evolution of the *LEGCYC1B* lineage, which is closely associated with floral morphological changes in *Lupinus*. Papilionoideae have strongly bilaterally symmetrical flowers, whereas *Cadia purpurea* flowers show radial symmetry associated with the expression of two *CYC* homologous genes (*LEGCYCs*) in the dorsal region of the flower (Figure 2) [58]. In addition, the expression pattern of one gene has expanded from the paraxial to the lateral and posterior regions of the corolla, which may result in reversion to evolutionarily regressive petal characters.

Wang et al. determined that the expression of three endogenous *LjCYC* genes is specifically inhibited by different RNAi transgenes [62]. A chimeric RNAi transgene containing *LjCYC1*- and *LjCYC2*-specific sequences down-regulated the expression of both endogenous genes. The effect of silencing the three *LjCYC* genes was mainly confined to the dorsal or lateral part of the petals, implying that the genes are associated with dorsal and lateral activities during the development of zygomorphic flowers [62]. Knockdown of the three *LjCYC* genes may result in wild-type petals that resemble ventral petals, complete organ internal (IN) asymmetry, and the lack of dorsoventral (DV) pathway-differentiated flowers. This suggests that DV asymmetry during the development of zygomorphic flowers is controlled by *LjCYC* genes, whereas floral organ IN asymmetry is independently determined by other genetic factors.

The mutation of *CYC2* in *Lathyrus odoratus* causes a change in dorsoventral petal type, resulting in a hooded (hdd) flower mutant with an epidermis and the pigmentation characteristic of a wing petal, and with a concave standard petal, the same as the lobed standard (*lst1*) mutant in *Pisum* [63]. Differences in *CYC* expression and activity may lead to differences in dorsal petal morphology in *Fabaceae,* and play a role in the negative regulation of petal edge growth in *Lathyrus*, mainly maintaining the flatness of the dorsal petal [64]. Interestingly, Ojeda et al. found that changes in the timing of *LjCYC2* expression during pollination of *Lotus* by bees and birds may be responsible for changes in flower petal micromorphology and size, whereas changes in the spatial distribution of gene expression had no effect on pollination [65].

Feng et al. determined that the upstream promoter regions of *GmCYC* genes vary in number and type of hormone response elements in *Glycine max* [66]. The expression of *GmCYC* genes is involved in different growth and developmental stages, induced by abscisic acid, brassinosteroids, aminocyclopropane–1–carboxylic acid, salicylic acid, and methyl jasmonate signals [66]. The *CYC*-like genes may have undergone multiple duplications and losses in different *Fabaceae* lineages and formed the distinct homologous clades CYC1 and CYC2, but the CYC3 clade was most likely lost [67]. The ancestors of *Papilionoideae* and *Caesalpinioideae* probably possessed two *CYC1* gene copies, but one of the copies was subsequently lost in *Papilionoideae* and was retained only in a few species of *Caesalpinioideae* [67]. The *CYC2* gene was replicated more frequently in *Papilionoideae* than in other legumes [67]. The diversity patterns of *CYC1* and *CYC2* genes are not associated with floral symmetry in non-papilionoid legumes, but the replication and functional differentiation of *CYC2* genes is necessary for floral symmetry in *Papilionoideae* [67].

The expression pattern of *VrCYC3*, which is homologous to *L. japonicus LjCYC3* and pea *PsCYC3*, differs from that of *VrCYC1* and *VrCYC2* in the dorsal, lateral, and ventral petals in mung bean (*Vigna radiata*) [68]. In addition, VrCYC3, which is localized to the nucleus, can induce transcription [68]. Moreover, it can interact with VrCYC1 and VrCYC2 in yeast cells, but this interaction is weakened by the deletion of two amino acid residues in its R domain [68]. This suggests that *LjCYC3*/*PsCYC3*/*VrCYC*3 play a conserved role in determining the lateral petals shape, and the formation of symmetrical and asymmetrical flowers in *Fabaceae*.

## 3. Progress in Research on *CYC*-like Genes in *Asteraceae*

*Asteraceae* is the most highly evolved family of dicotyledonous plants with a complex inflorescence structure, termed a capitulum, that often consists of radially symmetrical disc florets and bilaterally symmetrical ray florets [69,70]. Researchers have cloned snapdragon *CYC* homologs in *Helianthus*, *Gerbera*, *Senecio*, *Chrysanthemum*, and other genera, which revealed the considerable abundance of these homologs in *Asteraceae*, many of which have diverse functions [71,72,73,74,75]. At the single-floret level, the *CYC* gene in the *Asteraceae* species uniquely regulates the elongation of the corolla limb of ray florets, which are critical for attracting pollinators [76].

### 3.1. CYC-like Genes of Helianthus

The radiate sunflower (*Helianthus annuus*) capitulum consists of bilaterally symmetrical sterile ray florets and radially symmetrical bisexual disc florets. Ten ECE clade members have been identified in the sunflower, and the spatiotemporal expression of these homologous genes varies [77]. The specific temporal expression of the different genes in diverse plant parts, including ray florets, disc florets, leaves, and roots, may maintain the complex sunflower inflorescence structure via coordinated expression.

The sunflower *tubular ray flower* (*turf*) mutant has hermaphroditic ray florets with an almost actinomorphic corolla. Fambrini et al. determined that this mutation was caused by the insertion of a TCP motif, a non-autonomous transposable element (TE) from the *CYC*-like gene *HaCYC2c*, named *Transposable element of turf1* (*Tetu1*) [78]. The excision of *Tetu1* can restore the wild-type phenotype or produce stable mutants, indicating that *HaCYC2c* is a key regulator of ray floret symmetry. A loss-of-function mutation to *HaCYC2c* can promote the transition of sterile florets to hermaphroditic florets, which reflects the importance of *CYC*-like genes for the inhibition of stamen development.

**Figure 3:**
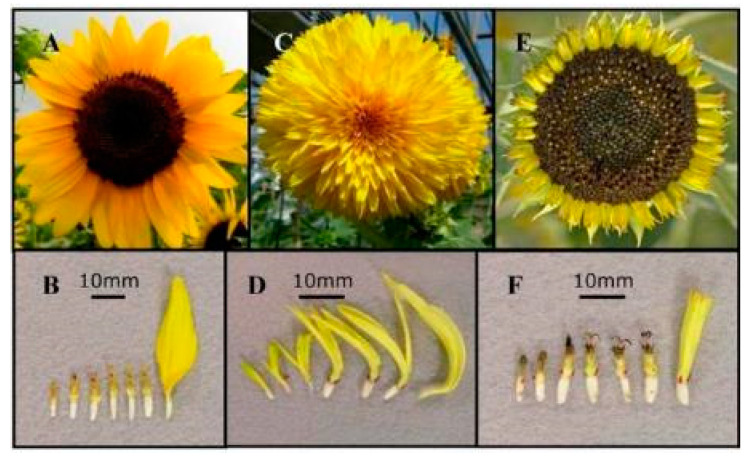
Capitulum and florets of a wild-type sunflower plant and two mutants [79]. (**A**,**B**) Wild-type sunflower with disc and ray florets. (**C**,**D**) *dbl* mutant with disc and ray florets. (**E**,**F**) *tub* mutant with disc and ray florets. The florets from the disc center to the peripheral florets are arranged from left to right. Reprinted with permission from Ref. [79]. Copyright © 2023 Chapman et al.

*HaCYC2c* was mutated in two independent *tubular-rayed* (*tub*) mutants, which apparently involved TE insertions, resulting in little or no expression and the formation of radially symmetrical ray florets, which are usually bilaterally symmetrical [79]. If *HaCYC2c* was inserted into the offspring, ray florets were more likely to replace disc florets at the center of the capitulum, whereas if *HaCYC2c* expression was inhibited, bilaterally symmetrical ray florets did not develop, and the capitulum comprised only disc florets (Figure 3).

Thus the *turf* and *tub* mutants are characterized by a transition from bilaterally symmetrical to radially symmetrical ray florets because of the insertion of TEs in *HaCYC2c* [80]. In the *dbl* or *Chrysanthemoides* (*Chry*) mutants, the insertion of *HaCYC2c* upstream of the coding region results in the ectopic expression of this gene and a transition from radially symmetrical disc florets to bilaterally symmetrical disc florets. The loss-of-function mutation to the *CYC*-like gene in sunflower *turf* mutants reportedly results in hermaphroditic tubular-like florets, which replace the normal sterile ray florets and the formation of a capitulum type that is not normally found in sunflower [81].

*HaCYC2c* was overexpressed after an insertion into the *HaNDUA2* promoter region to generate the sunflower long petal mutant (*lpm*) in which the abnormal elongation of the disc floret corolla and stamen abortion at an early stage of floral organ development was observed [82]. Furthermore, the floret symmetry changed from radial symmetry to bilateral symmetry, thus transforming the disc florets into ray florets. The overexpression of *HaCYC2c* and its control of *HaNDUA2* through transcriptional recognition may be an important regulatory node for floret type and functional differentiation in *Helianthus*, which was associated with maintaining the balance between the pollinator recruitment ability and the fertility of disc florets [82].

*HdCYC2c* and *HxmCYC2cB* belong to the CYC2 subclade in *Helianthus* [83]. *HdCYC2c* was differentially expressed in the different floret types of *Helianthus decapetalus*, with the expression level higher in the ray floret corolla than in the disc floret corolla [83]. In *Helianthus* × *multiflorus*, the insertion of TEs in *HxmCYC2cB* promoted the ectopic expression of *HxmCYC2cB* throughout the inflorescence, leading to the observed loss of actinomorphic florets and the production of ray florets [83]. Removal of a TE (CTEHM1) and epigenetic regulation of *HmCYC2c* expression resulted in two capitulum types of *Helianthus* × *multiflorus*, Meteor 1 and Meteor 2 [37]. The expression of *HmCYC2c* in the disc floret of Meteor 2 was significantly higher than that of Meteor 1. The CTEHM1 in *HmCYC2c* was truncated in Meteor 1, which showed the typical tubular corolla of *Helianthus*, whereas the remaining presence of CTEHM1 in *HmCYC2c* of Meteor 2 caused the largest corolla of disc florets to display the characteristics of a ray floret, resulting in an entirely radiate capitula not normally found in *Helianthus* [37].

Fambrini et al. isolated three CYC2 subclade genes (*HrCYC2c*, *HrCYC2d*, and *HrCYC2e*) associated with the identity of the *Helianthus* ray–floret (Hr), among which *HrCYC2c* played an important role in the initiation of the ray floret primordium [38]. The capitula of *HrCYC2c*-mutant homozygous dominant plants (*HrCYC2c*/*HrCYC2c*) and heterozygous dominant plants (*HrCYC2c*/*HrCYC2c-m*) initiated ray florets, whereas the recessive homozygous plants (*HrCYC2c-m*/*HrCYC2c-m*) did not develop ray florets [38].

### 3.2. CYC-like Genes of Gerbera Hybrida

**Figure 4:**
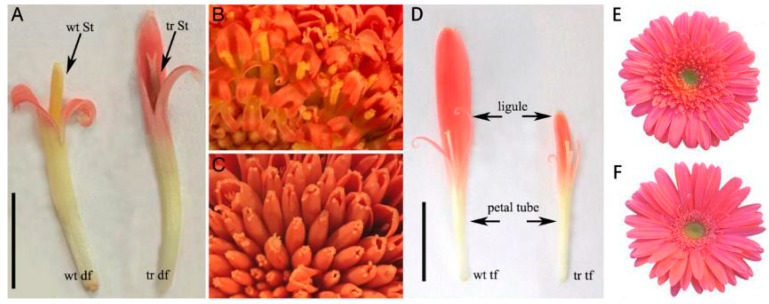
*G. hybrida* with *GhCYC2a* overexpression (**A**–**C**) and inhibition (**D**–**F**) [71]. (**A**) Disc florets (df) of wild-type (wt) *Gerbera hybrida* and transgenic (tr) *G. hybrida* with obvious phenotypic differences. St, stamen. (**B**) Pollen presentation on the style of the disc florets of wild-type *G. hybrida*. (**C**) Disc florets of transgenic *G. hybrida* lacking functional stamens. (**D**) Transitional floret (tf) of wild-type and transgenic *G. hybrida*. (**E**) Capitulum of wild-type *G. hybrida*. (**F**) Capitulum of genetically modified *G. hybrida*. Reprinted with permission from Ref. [71]. Copyright © 2023 by The National Academy of Sciences of the USA.

The *CYC*-like homolog *GhCYC2a* is involved in the differentiation of *Gerbera hybrida* floret types, and its expression exhibits a gradient along the radial axis of the capitulum [71]. Specifically, *GhCYC2a* is expressed in the peripheral, bilaterally symmetrical ray florets, but not in the centermost disc florets, which are almost radially symmetrical and have more deeply incised corolla lobes. The overexpression of *GhCYC2a* results in disc florets acquiring a morphology similar to that of ray florets, whereas the inhibition of *GhCYC2a* expression leads to the development of limbs that are shorter than those of wild-type ray florets (Figure 4). This provided the first molecular evidence that a *CYC*-like TCP TF is involved in the definition of the capitulum of the *Asteraceae* species.

*GhCYC2a* collaborates with other *CYC*-like genes to participate in floret differentiation and ultimately determine the complex capitulum structure of *G. hybrida* [84]. *GhCYC2a* is specifically expressed in ray florets at an early developmental stage and is only activated in tubular flowers at an advanced developmental stage. *GhCYC2b* in *G. hybrida* and *HaCYC2d* and *HaCYC2c* in sunflowers belong to the CYC2 clade considered to be a strong candidate as regulators of ray–floret identity [74]. When *GhCYC2b* expression was inhibited in *G. hybrida*, the third type of transitional florets were shorter and the ray–floret corolla was five- or eight-lobed and radially symmetrical, which was in accordance with the findings of Broholm [84].

There is a substantial overlap in expression patterns among the CYC2 subclade genes (i.e., *GhCYC2a*, *GhCYC2b*, *GhCYC2c*, and *GhCYC2d*) in *G. hybrida* [74]. At the single-floret level, their expression domains in the corolla shifted spatially from the currently known dorsal pattern in bilaterally symmetrical flower species, which may have evolved after the origin of *Asteraceae* [74]. *GhCYC2a*, *GhCYC2b*, and *GhCYC2c* mediate the positioning in the proximal and distal axes of the capitulum, leading to ray floret differentiation, and also regulate ray–floret corolla growth by affecting cell proliferation until the corolla assumes its final size and shape [74]. In contrast, the expression of *GhCYC2d* may increase the floret initiation rate during the expansion of the capitulum, while the ectopic expression of *GhCYC2d* increases the floret density in the capitulum [74]. The upstream regulators of *GhCYC2b* (i.e., GhCIN1 and GhCIN2) are CINCINNATA-like homologous TCP proteins with unknown expression domains and functions, but are known to delay the development of marginal ray–floret primordia during early ontogeny [85]. In developing ray florets, the class E MADS-box TF GRCD5 activated *GhCYC2b* expression, whereas the class C MADS-box TF GAGA1 (upstream of *GhCYC2b*) contributed to stamen development.

### 3.3. CYC-like Genes of Senecio

Natural polymorphism of the capitulum in the *Senecio* species is due to the transfer of a set of regulatory genes containing the *RAY* locus from the diploid *Senecio squalidus* to the tetraploid *Senecio vulgaris* [86]. The *RAY* locus, which comprises a cluster of *CYC*-like genes expressed in the periphery of the inflorescence meristem that promote floral asymmetry and lead to increased outcrossing rates, has played a key role in the evolution of radiate capitulum types. The *CYC2*-like gene *RAY3* is initially uniformly expressed in ray florets during capitulum development, but at advanced stages is expressed only in the ventral corolla lobes of ray florets, resulting in the elongation of the ventral corolla limb in *S. vulgaris* [72]. The diversification of *CYC*-like genes has led to novel interactions, with *SvDIV1B* inhibiting *RAY3*, but potentially activating *RAY2*. The expression of *SvRAY1* may induce lateral cell division during the development of the *S. vulgaris* ray floret and, as a result, the morphology and arrangement of the ray floret cells change to some extent, thereby affecting the ray floret width [87]. The ray florets of *SvRAY1*-overexpressing plants were shorter and significantly broader than the wild-type ray florets.

### 3.4. CYC-like Genes of Chrysanthemum

Researchers have cloned the homologs of snapdragon *CYC* genes in several *Chrysanthemum* × *morifolium* cultivars and analyzed their expression and function [88,89,90,91,92]. Huang et al. identified six *CYC2* subclade *CmCYC2* genes (i.e., *CmCYC2a*, *CmCYC2b*, *CmCYC2c*, *CmCYC2d*, *CmCYC2e*, and *CmCYC2f*) in the *C.* × *morifolium* cultivar ‘Maoxiangyu’, wherein they mainly regulate the development of ray florets [88]. Compared with other *CYC* homologs, *CmCYC2*s in chrysanthemum may be similarly expressed or there may be distinct differences in expression patterns. The overexpression of *CmCYC2d* in wild-type *Arabidopsis thaliana* and the *tcp1* mutant showed that the vegetative growth of the transgenic lines was inhibited, the flowering period was delayed, and the petal size and arrangement were changed, making the originally radially symmetrical petals appear bilaterally symmetrical [88]. Furthermore the CmCYC2 proteins may form homodimers during flower organogenesis and participate in the regulation of ray and disc floret morphogenesis.

Chen et al. observed that the spatial expression patterns of six *Asteraceae CYC2*-like members are conserved throughout the family, and all of them influence capitulum development [93]. Both *CYC2c* and *CYC2g* are important for ray floret formation in *Chrysanthemum lavandulifolium*, whereas *CYC2d* inhibited the development of the dorsal corolla lobes and ray–floret stamens. The class A MADS-box genes interacted with *CYC2*-like genes potentially involved in processes associated with the formation of reproductive organs and the ray–floret corolla, especially corolla differentiation of the disc and ray florets in chrysanthemum [94]. The class B MADS-box gene *CDM19* may positively regulate the expression of the *CYC2*-like genes *CmCYC2c* and *CmCYC2d*, thereby modifying the floret symmetry in chrysanthemum [95].

Yang et al. cloned the *C.* × *morifolium* gene *CmTCP7*, which may be involved in the formation of floral buds as well as promote the growth of the corolla of the ray floret and participate in the formation of bilaterally symmetrical ray florets [96]. The *CYC*-like gene *CmCYC2* and *WUS*-like gene *CmWUS*, which were highly expressed in floral buds at the time of floral organ differentiation and in reproductive organs at advanced stages of development, coordinately regulate the development of *C.* × *morifolium* reproductive organs [89]. Furthermore, *CmCYC2* was highly expressed in the corolla of ray florets, which may promote ray floret growth and contribute to the formation of bilaterally symmetrical ray florets.

The expression of *CYC2b*, *CYC2d*, *CYC2e*, and *CYC2f* was differentially expressed in different types of *Chrysanthemum vestitum* ray florets, which confirmed the influence of *CYC*-like genes on floral morphology [90]. Yuan et al. observed that the ectopic expression of *CmCYC2* in the *Arabidopsis tcp1* mutant altered flower symmetry and flowering time, and the CmCYC2 TF may interact with or bind to the *CmCYC2* promoter to regulate floral symmetry development in *Chrysanthemum* [91]. Liu et al. cloned the *CYC2*-like gene *Cyc2CL* from *C.* × *morifolium* ‘Pink Carpet’ and revealed for the first time the variable shear pattern of a *CYC2*-like gene in chrysanthemum [92]. The transcription of *Cyc2CL* resulted in two mature mRNA sequences (*CyC2CL-1* and *CyC2CL-2*). Both transcripts were present at high levels in ray florets, but at very low levels in disc florets and inhibited the development of petals and stamens in *A. thaliana*.

**Figure 5:**
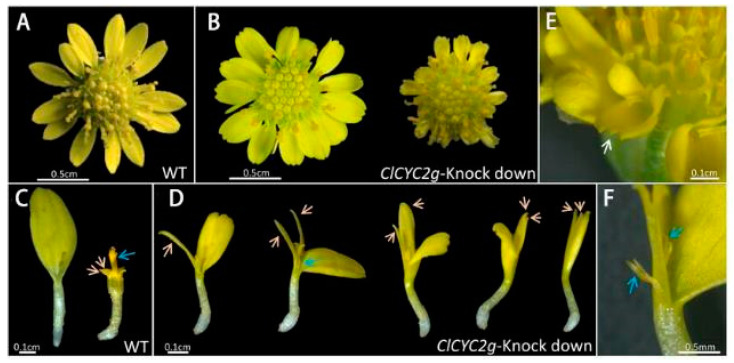
Knockdown of *ClCYC2g* expression adversely affects the formation of symmetrical marginal florets in the radiate capitulum in chrysanthemum [97]. (**A**,**B**) Morphology of the capitulum of wild-type (WT) and transgenic *C. lavandulifolium*. (**C**) Ray and disc florets of wild-type *C. lavandulifolium*. (**D**) Ray-shaped florets with gradually increasing mutations. Orange and blue arrows indicate abnormal petals and stamens, respectively. (**E**,**F**) Expanded marginal florets of the transgenic lines. White and blue arrows indicate the bilabiate corolla with a deeply dentate limb apex and stamens, respectively. Reprinted with permission from Ref. [97]. Copyright © 2023 Society for Experimental Biology and John Wiley & Sons Ltd. (Hoboken, American).

The morphogenesis of the marginal florets in *Ajania* is interrupted shortly after the formation of the floral primordia, possibly because of the lack of expression of the *CYC2*-like gene *ClCYC2g* [97]. The decreased expression of *ClCYC2g* in *C. lavandulifolium* results in the gradual transformation of ray florets into disc florets (Figure 5). This transition may be associated with changes in pollination strategies under selective pressure. Zhang et al. identified four *ClCYC2*-like genes (i.e., *ClCYC2c*, *ClCYC2d*, *ClCYC2e*, and *ClCYC2f*), for which expression levels were significantly higher in ray florets than in disc florets of *C. lavandulifolium* [98].

### 3.5. CYC-like Genes of Other Asteraceae Groups

Bello et al. recovered eight major gene lineages in the highly derived genus *Anacyclus* (tribe *Anthemideae*) through phylogenetic reconstruction, comprising two *CYC1* genes, four *CYC2* genes, and two *CYC3* genes [21]. In *Anacyclus*, three *AcCYC2* genes are highly expressed in ray florets, and the expression patterns of four *AcCYC2* genes overlap in multiple organs, including the limb of ray florets, anthers, and ovule throughout development. Gene duplication events, as well as the subsequent subfunctionalization and neofunctionalization of *SEPALLATA*-like genes and *CYC*-like genes in *Asteraceae,* have been shown to be conducive to the identification of the floral meristem and the formation of key traits for floral differentiation in this large family [99]. Sun et al. identified five *CYC2*-like genes in several *Gaillardia* cultivars with different ray floret types [73]. Analyses of RNA re-sequencing results, quantitative real-time PCR (qRT-PCR) data, and the effects of gene silencing suggested that *CYC2c* is the main genetic factor affecting the formation of ray florets in *Gaillardia*.

## 4. Progress in Research on *CYC*-like Genes in *Lamiales*

Gene duplication, gene family retention, and tissue-specific expression of *CYC*-like genes are believed to have affected the evolution of corolla symmetry in *Lamiales* [100,101]. The *CYC*-like genes were differentially expressed in the higher core clades with high expression levels in adaxial petals, which had been widely replicated in *Lamiales* (including *Lamiaceae*, *Scrophulariaceae*, *Gesneriaceae*, *Oleaceae*, *Phrymaceae*, and many other families) [102,103]. The asymmetrical expression of *CYC*-like genes was not common but associated with the origin of bilaterally symmetrical corollas [104]. Changes to the *cis*-regulatory domain and the coding sequence of *CYC*-like genes may be critical for the symmetrical evolution of both sides of the corolla, with multiple selection mechanisms contributing to gene retention [100].

The expression pattern of *CYC2*-like genes has gradually evolved, and was widely expressed in the meristem of early-diverging *Lamiales* with a bilaterally symmetrical corolla, but limited in the meristem of core *Lamiales* and thus may be related to the origin of corolla bilateral symmetry [105,106]. The repeated loss of bilateral corolla symmetry is relatively frequent in *Lamiaceae*, which may be caused by different mechanisms and changes in floral symmetry-related genes, such as the loss of the CYC2 clade gene *Ml-CYC2A* in the genome and the contraction, expansion, or altered expression of *Cc-CYC2A* [107,108]. Sengupta and Hileman detected the significant enrichment of predicted autoregulatory sites in the 5′-terminal upstream noncoding region of *CYC*, the upstream regulator of floral zygomorphy in *Lamiales*. Their results suggest that the correlation between the autoregulation of *CYC* and the origin of zygomorphic flowers may be associated with zygomorphic flowers independently derived from eudicot lineages [40].

### 4.1. CYC-like Genes of Scrophulariaceae

*Scrophulariaceae* inflorescences are typically racemose, spicate, or cymose and often form a panicle [109]. The *CYC* gene associated with the regulation of floral symmetry was initially isolated from snapdragon, and its homologs in related species were subsequently cloned, including *Linaria vulgaris*, *Mohavea confertiflora*, *Veronica montana*, *Gratiola officinalis*, and *Torenia fournieri* [1,110,111,112,113,114]. These genes have diverse functions in *Scrophulariaceae*, but they primarily affect the morphological characteristics of petals and the development of stamens.

**Figure 6:**
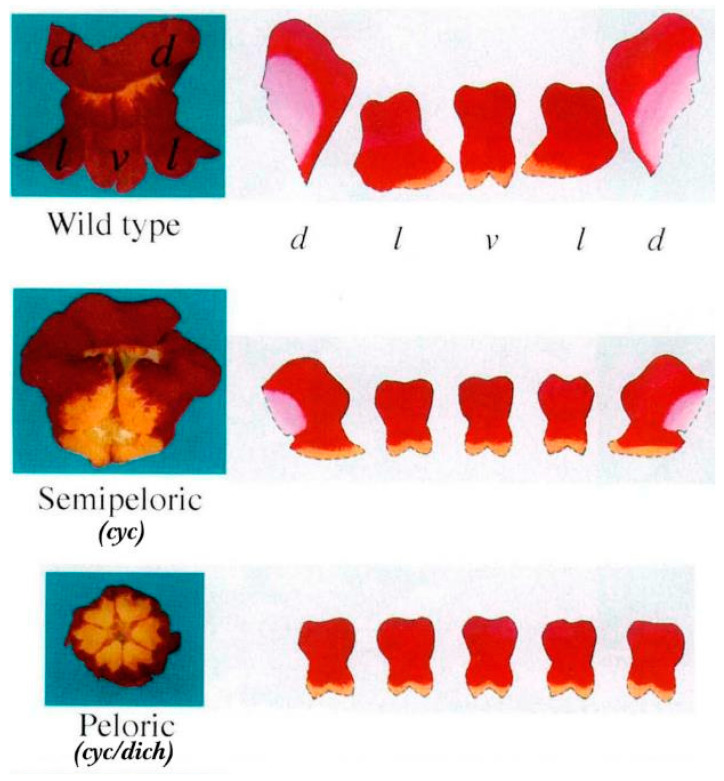
Flowers of wild-type and mutant snapdragon plants [1]. Photographs of the dorsal corolla lobe (d), lateral corolla lobe (l), and ventral corolla lobe (v) of the wild-type snapdragon flower are presented. The characteristics of the different corolla lobes are shown to the right of each flower. Reprinted with permission from Ref. [1]. Copyright © 2023, Nature Publishing Group.

The wild-type snapdragon corolla comprises two dorsal lobes, one ventral lobe, and two lateral lobes. Snapdragon mutants have a semi-abnormal regular flower (semipeloric; *CYC* mutation) or an abnormal regular flower (peloric; *CYC*/*DICH* double mutation), which represent bilaterally symmetrical floral transitions into a radially symmetrical flower (Figure 6) [1,115]. In the classic *DICH* homozygous mutant, the ventral corolla lobes are more symmetrical than the wild-type ventral lobes and usually separate from each other because of a deep incision between the dorsal lobes [1].

Corley et al. determined that *AmCYC* was expressed in the dorsal corolla lobe, in which *AmRAD* was activated [116]. This ultimately led to the inhibited expression of *AmDIV* in the ventral and lateral lobes, and the formation of asymmetrical snapdragon flowers. Li et al. revealed that whole-genome duplication (WGD) and tandem replication had contributed to the expansion of the *CYC* gene family [15]. Both (*RAD*) and (*DIV*) controlled floral symmetry downstream of *CYC*/*DICH* and interacted with *DIV-RAD-INTERACTING FACTOR* (*DRIF*) [15]. The *DRIF* genes, which had homologous copies similar to *CYC*/*DICH*, were also located in the WGD-derived syntenic block [15]. These results further support the view that the key genetic factor regulating the asymmetry of snapdragon flowers was the result of a WGD event.

The peloric flowers (i.e., transitional from bilateral symmetry to radial symmetry) of an *L. vulgaris* mutant were the result of a spontaneous epigenetic mutation to the *CYC* allele [110,117]. The mutant harbored a defective *LCYC*, which is a *CYC* homolog. *LCYC* underwent a heritable modification (i.e., extensive methylation and transcriptional silencing) that was co-isolated with the mutant phenotype [117]. The mutant phenotype may be reversed during somatic development, which is associated with the demethylation of *LCYC* and the restoration of gene expression.

Hileman et al. identified the *M. confertiflora CYC* and *DICH* homologs, *McCYC1*, *McCYC2*, *McDICH1*, and *McDICH2*, of which expression levels increase from the stamen to the outermost floral whorl, which may be due to the change in the expression domain of the regulatory genes in the *CYC/DICH* pathway [111]. Changes to the *McCYC* and *McDICH* expression patterns result in new floral morphological traits, in that the two lateral stamens are aborted and show evidence of the adaxial corolla lobes’ internal symmetry. The expression of *CYC*-like genes led to delayed growth or degradation of the adaxial floral organs, but it may also be associated with the loss of the adaxial floral organs [1,111].

A conservative floral symmetry gene network exists in *V. montana* and *G. officinalis*, in which *CYC*-like genes evolved after the gene duplication event, although the detailed genetic mechanisms of dorsal and ventral stamen abortion differ [114]. Specifically, *VmCYC1*, *GoCYC1*, and *GoCYC2* are only expressed in the dorsal region of the floral meristem and in developing flowers, in which expression patterns are independent of stamen abortion patterns, whereas the expression of *VmCYC2* and *GoCYC3* is mainly detected in vegetative and floral tissues.

A dorsally expressed *CYC*-like gene and the downstream target genes *RAD* and *DIV* are absent in *Plantago major* but are present in *Aragoa abietina* [53]. This *CYC*-like gene is expressed in all parts of the flower in *A. abietina*, including the dorsal, ventral, and lateral regions, similar to the expression of its homolog in the related species *Veronica serpyllifolia*. The duplication of *CYC*-like genes led to the evolution of radially symmetrical *A. abietina*/*P. major* flowers, and further disintegration of the symmetrical flower-related gene pathway led to the wind-pollination syndrome of *P. major* [66]. This model emphasizes the potential importance of gene loss in the evolution of important ecological traits.

Su et al. detected recent replication events of a *CYC*-like gene in *T. fournieri*, and functional analysis of two genes that show dorsal-specific expression, *TfCYC1* and *TfCYC2*, suggested the existence of a regulatory module integrating the dorsoventral pattern and asymmetric corolla pigmentation [112]. The ectopic expression of *TfCYC2* disrupts the asymmetrical corolla coloring pattern, resulting in a strongly dorsal flower, and the CYC–RAD module coordinates petal shape and corolla pigmentation. When *TfCYC2* expression was downregulated, the dorsal petal identity was lost. Diversified *CYC* genes evolved regulatory loops, and *TfCYC2* was directly bound to the regulatory region of the R2R3-MYB gene *TfMYB1*, resulting in asymmetric expression and ultimately the establishment of asymmetric pigmentation patterns [112]. Integration of the Ty1/Copia-like LTR retrotransposon TORE2 into the exon of *TfCYC2*, to generate the allele TfCYC2^TORE2^, inhibited the expression of *TfCYC2*, which is the main regulatory gene involved in anthocyanin pattern enrichment in *T. fournieri* [118]. The degree of pigmentation of the dorsal corolla lobe of *T. fournieri* is negatively correlated with *TfCYC2* expression.

### 4.2. CYC-like Genes of Gesneriaceae

The inflorescences of the *Gesneriaceae* species are usually double-flowered cymes or monochasia. The flowers are usually bilaterally symmetrical, but some species produce radially symmetrical flowers (e.g., *Tengia scopulorum*). In recent years, *CYC*-like genes have been isolated from several members of this family, including *Saintpaulia ionantha*, *Sinningia speciosa*, *Chirita heterotricha*, *Primulina heterotricha*, and *Petrocosmea glabristoma* [54,57,119,120]. These genes have the typical functions of *CYC*-like genes, which affect floral symmetry and stamen abortion.

**Figure 7:**
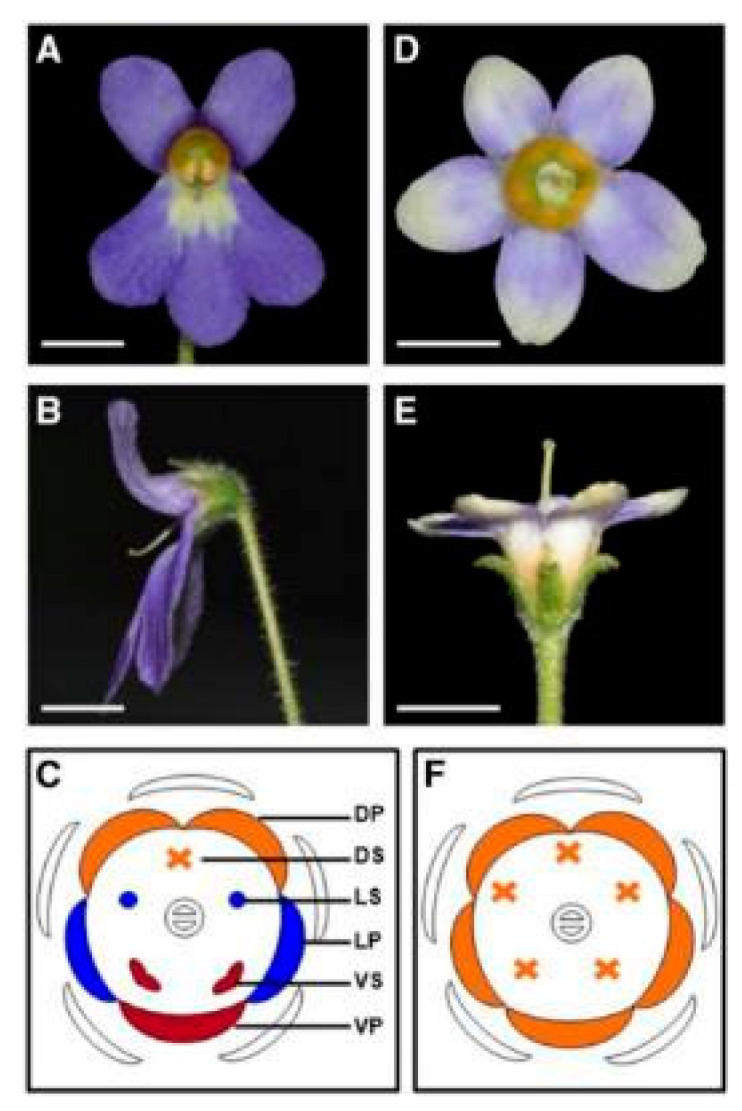
Front and side view and floral diagram of normal and peloric flowers of *Petrocosmea sinensis* [54]. (**A**–**C**) Front and side view and floral diagram of *P. sinensis* normal flowers, which have a typical bilaterally symmetrical corolla. (**D**–**F**) Front and side view and floral diagram of *P. sinensis* peloric flowers, which have a radially symmetrical corolla. DP, dorsal corolla lobes; DS, dorsal stamens; LS, lateral stamens; LP, lateral corolla lobes; VS, ventral stamens; VP, ventral corolla lobe. Reprinted with permission from Ref. [54]. Copyright © 2023, Oxford University Press.

The successive examination of the *CYC*-like genes in the *C. heterotricha*, *P. heterotricha*, and *Petrocosmea* species showed that their expression in developing flowers is regulated by various mechanisms [54,119,120]. First, the promoter sequences of the *CYC* homologs *ChCYC1C* and *ChCYC1D* in *C. heterotricha* were isolated. Subsequent analysis indicated the genes may have evolved automatic regulatory loops to maintain expression during the establishment of bilaterally symmetrical flowers [119]. The *RAD*-like gene *ChRAD* may be directly targeted by *ChCYC1* as part of a regulatory network. Next, the expression and function of two *CYC2* genes (*CYC1C* and *CYC1D*) in *P. heterotricha* were analyzed, which revealed positive self-regulatory and cross-regulatory effects [120]. This mechanism may lead to the independent formation of bilaterally symmetrical flowers, which is associated with plant–insect co-evolution and the adaptive radiation of angiosperms. Finally, changes in the dorsal corolla lobe size of *P. glabristoma* and *Petrocosmea sinensis* were determined to be mainly mediated by the expression and differentiation of *CYC1C* and *CYC1D*, and the changes in the petal shape were associated with the expression-level changes to the *CIN*-like TCP gene *CIN1* (Figure 7) [54]. Highly redundant homologous genes with the same expression patterns and interspecific differences in expression may be controlled by markedly different regulatory pathways, because natural selection may have resulted in diverse regulatory modifications rather than sequence changes to key developmental genes to generate morphological diversity [54].

Hsu et al. reported that in *S. speciosa*, the dorsal corolla lobes are bent outward, the midvein of the lateral corolla lobes is asymmetrical, and the expansion of the ventral area of the corolla is closely related to the *SsCYC* genotype [57]. Expression shifts of the *CYC*-like genes *SiCYC* and *SiCYC1B*, which show dorsal-specific expression in the wild-type *S. ionantha*, led to two completely different reversals of radial symmetry, namely dorsalized actinomorphic (DA) and ventralized actinomorphic (VA) peloria, which may be controlled by upstream *trans*-acting factors or epigenetic regulation [121]. *SiCYC* and *SiCYC1B* were metastasized with an ectopically extended expression on all corolla lobes in DA, whereas their dorsal-specific expression was greatly reduced in VA [121]. The main highly expressed copies of *SiCYC* were constrained by purification selection, whereas selection of the low-expression helper gene *SiCYC1B* was relaxed after duplication [121]. Heterologous expression of *SiCYC* in *A. thaliana* was characterized by delayed corolla growth owing to limited cell proliferation [121].

*CYC*-like gene duplication events have occurred at least five times in the evolutionary history of *Gesneriaceae* [51]. Three copies of *CYC*-like genes in the actinomorphic *Conandron ramondioides* were not expressed in the corolla, whereas the zygomorphic species *Hemiboea bicornuta* and *Lysionotus pauciflorus* retained a *CYC1* copy (i.e., *GCYC1C* and *GCYC1D*, respectively) expressed in dorsal corolla lobes [51]. Selective relaxation after the duplication of *CYC1* created evolutionary diversification, in which multiple copies retained the effect of random differentiation affecting the dorsal-specific expression of genes associated with floral symmetry changes [51]. The promoter region of *CpCYC* is a key determinant of its specific expression in the dorsal corolla lobe of *Chirita pumila*, where the LEAFY element may directly activate and regulate *CpCYC* to form a bilaterally symmetrical flower [122].

Yang et al. determined that the ortholog of *LjCYC1* in *S. ionantha* is highly expressed in the root, leaf, peduncle, calyx, petal, stamen, and pistil of transgenic *S. ionantha* plants [123]. Two flower-type variations were observed in T_1_ transgenic plants. The first was the change in floral symmetry. Specifically, radially symmetrical wild-type flowers were replaced by bilaterally symmetrical flowers or flowers with obvious differences between the dorsal and ventral corolla lobes. The second variation involved floral organ morphology (e.g., a lobe incision towards the base of the corolla, and stamen, pistil, and calyx petalization). Liu et al. functionally characterized the *CYC*-like flower symmetry-related gene *CpCYC* in *C. pumila* [124]. By transforming plants with a RNAi:CpCYC vector, vertically radially symmetrical flowers were obtained, implying that *CpCYC* determines the establishment of zygomorphy and the horizontal plane of flowers. The insertion of a *CpCYC* promoter:GUS vector into *C. pumila* confirmed that the *CpCYC* promoter was active in dorsal corolla lobes, dorsal/lateral staminodes, and pedicels.

### 4.3. CYC-like Genes of Phrymaceae

The *Phrymaceae* species have bilaterally symmetrical bisexual flowers borne in spikes at the top of the stem and in the upper leaf axils. To date, there has been relatively little research on the *CYC*-like genes in this family. The flowers of the *Phrymaceae* species *Diplacus pictus* have distinct dorsal, ventral, and lateral corolla lobes. The expression and function of *CYC* genes may vary between *D. pictus* and snapdragon [125]. The *CYC*-like gene *DpCYC* is expressed in a narrow part of the upper lip of the dorsal corolla lobe. The novel upturned abaxial corolla lobe of *D. pictus* may be associated with the localized expression of *DpCYC* on the upper surface of this structure.

## 5. Progress in Research on *CYC*-like Genes in *Orchidaceae*

*Orchidaceae* is a large family, second in species number only to *Asteraceae* and the largest family of monocotyledons [126,127]. The bisexual flowers of the members of this family are generally bilaterally symmetrical [128]. The median tepal is typically modified into the labellum, which is often in the abaxial position because the inflorescence droops or the pedicel twists [129]. Many *CYC*-like genes in *Phalaenopsis* and *Cattleya trianae* have been identified and observed to influence floral or inflorescence development [130,131,132].

Lin et al. observed that the ECE clade gene *PeCIN8* is highly expressed in the late ovule developmental stage, with overlapping expression on the 16th day after the pollination of *Phalaenopsis equestris* flowers [130]. Hence *PeCIN8* may be crucial for orchid ovule development because of its regulatory effects on cell division. In addition, Liu et al. recently identified 10 ECE clade genes (*CgCIN*s and *CgCYC*s) from the *Cymbidium goeringii* genome and observed that flower-specific gene expression may be associated with the regulation of flower development [131].

The *CYC*-like genes are single, short, low-expressed copies in *Orchidoideae* [132]. Madrigal et al. performed a phylogenetic analysis of the TCP genes in *C. trianae* and observed that the TCP genes were resolved into three major clades with multiple gene duplication events detected [132]. Among these genes, the *CYC*-like genes are single short copies with low expression, and their homogeneous expression in the labial and lateral tepals suggested that they contribute little to bilateral perianth formation.

## 6. Progress in Research on *CYC*-like Genes in *Solanaceae*

The *Solanaceae* is dominated by species with radially symmetrical flowers, but the early-diverging clades often show bilateral flower symmetry [133]. At present, few studies have explored the influence of *CYC*-like genes on flower development in the *Solanaceae*. *Petunia hybrida* is an annual herb that produces solitary flowers in leaf axils [134]. Its funnel-shaped corolla is white or purple and may be variously striped. There is considerable diversity in the flower types of *P. hybrida*. Research on *Petunia CYC*-like genes of the ECE clade showed that they may be mainly associated with the growth and development of axillary buds, while also modulating stem growth and the development of branches, flowers, and leaves (e.g., size) [135,136,137].

Zou et al. isolated the *P. hybrida CYC1* clade genes *PhTCP3* and *PhTCP4*, the *CYC2* clade gene *PhTCP2*, and the *CYC3* clade gene *PhTCP1*, which were predominantly associated with axillary bud growth and development [135]. These four genes were highly homologous to the ECE clade genes from tomato (*Solanum lycopersicum*), gerbera, grape (*Vitis vinifera*), poplar (*Populus*), *A. thaliana*, and other plant species and exhibited tissue-specific expression patterns, and the expression levels in the axillary buds were significantly higher than those in the other tissues analyzed [135]. The overexpression and silencing of *PhTCP1* led to decreased and increased branching, respectively, suggestive of the regulatory effects of *PhTCP1* on branch formation and development.

Zhang et al. conducted the first genome-wide spatiotemporal expression profile and promoter analysis of the petunia ECE clade *PaTCP* genes, and reported that these genes may play an important role in the various developmental processes of petunia through multiple hormonal pathways, especially in petal development and the formation of petal size [136]. Sengupta and Hileman observed that the *CYC* homologous genes positively regulate the *RAD* homologous genes in tomato, which was similar to previous findings in snapdragon [137].

## 7. Progress in Research on *CYC*-like Genes in Other Angiosperms

To date, there have been only a few studies on *CYC*-like genes in other angiosperm families. These investigations have indicated the genes are primarily expressed in floral organs and affect the establishment of floral symmetry and stamen fertility [138,139,140,141,142]. Thus future research needs to be extended to these less-studied plant families.

### 7.1. CYC-like Genes of Brassicaceae

The flowers of *Brassicaceae* are borne in racemes of many small flowers, which are actinomorphic in most species but zygomorphic in a few species [143,144]. Cubas et al. were the first researchers to identify a *CYC2* lineage gene, *AtTCP1*, in *A. thaliana* that regulates the symmetrical development of floral organs [52]. Arabidopsis flowers are actinomorphic, and *AtTCP1* is briefly expressed specifically in the dorsal region of the floral primordium, which suggests that *AtTCP1* does not play a key role in floral organ differentiation because there is no flower-specific direct transcriptional autoregulation or other expression pattern that persists until advanced stages of flower differentiation [52].

The corolla of the genus *Iberis* is zygomorphic, with two small adaxial petals and two large abaxial petals [145]. Busch et al. identified a *CYC* homolog from *Iberis amara*, *IaTCP1*, for which the temporary alteration of expression is important for the control of zygomorphic corolla formation [146]. The timing of *IaTCP1* expression differed from that of *AtTCP1* and other *CYC* homologs. Specifically, *IaTCP1* expression was lacking early in asymmetric petal development, but the gene was strongly differentially expressed in the corolla during advanced asymmetric petal development. In addition, *IaTCP1* activated the expression of many more genes in adaxial petals than it inhibited [147]. These findings suggest that asymmetric corolla formation in *I. amara* may be caused by the strong expression of the *CYC2* clade gene in the small ventral petals and weak expression in the large dorsal petals.

Busch et al. revealed through phylogenetic reconstruction that the zygomorphic genera *Iberis*, *Calepina*, and *Teesdalia* belong to a major *Brassicaceae* lineage [148]. Zygomorphy is most evident in *Iberis*, but less so in *Calepina* and *Teesdalia*, with an expression-dependent positive correlation between the strength of the difference in *CYC2* expression and the degree of zygomorphy [148]. This study suggested that zygomorphy evolved through the heterochronic expression of *CYC2,* from paraxial expression in the ancestral floral meristem to accumulation of paraxial *CYC2* transcripts late in petal development in the *Brassicaceae*.

### 7.2. CYC-like Genes of Dipsacales

Howarth and Donoghue identified three major ECE clade *CYC*-like genes (*DipsCYC1*, *DipsCYC2*, and *DipsCYC3*) in the ancestors of *Dipsacales,* as well as additional duplication events involving genes in this clade [26]. The *DipsCYC1* gene was not involved in subsequent replication events and may not be expressed in flower tissues. In contrast, *DipsCYC2* and *DipsCYC3* had similar duplication patterns in several clades. In the *Caprifoliaceae* species *Lonicera morrowii*, *DipsCYC2B* was expressed in the four dorsal petals, but not in the ventral petal, whereas *DipsCYC3B* was expressed in the flowers and petal primordia, with the peak expression level in the ventral petal [26].

Berger et al. compared the expression patterns of six *CYC*-like genes in the dorsal, lateral, and ventral petals of the inner and outer florets of the capitulum of *Knautia macedonica* and observed that *CYC*-like genes duplicated many times were differentially expressed among the petal types and the inner and outer florets [55]. The formation of bilateral symmetry may be regulated by a dorsoventral expression gradient. In addition, dorsoventral morphological specificity was associated with significant changes in ventral petal gene expression.

### 7.3. CYC-like Genes of Zingiberales

Bartlett and Specht showed that gene replication unique to the *Zingiberales*, including one replication event involving the ECE clade gene *TBL*, preceded the diversification of commelinid monocotyledons [142]. The changes in *TBL* expression were associated with the evolutionary changes in floral symmetry and stamen abortion. In addition, *ZinTBL1a* was expressed in the posterior (adaxial) stamen lip of *Heliconia stricta* (*Heliconiaceae*) and the anterior (abaxial) stamen lip of *Costus spicatus* (*Costaceae*) [141]. The *ZinTBL2* expression level was highest in the anterior sepals of *H. stricta* and the posterior fertile stamens of *C. spicatus*. These findings indicate that the ECE clade genes were repeatedly recruited in the evolutionary process, which accelerated the evolution of bilaterally symmetrical flowers.

**Figure 8:**
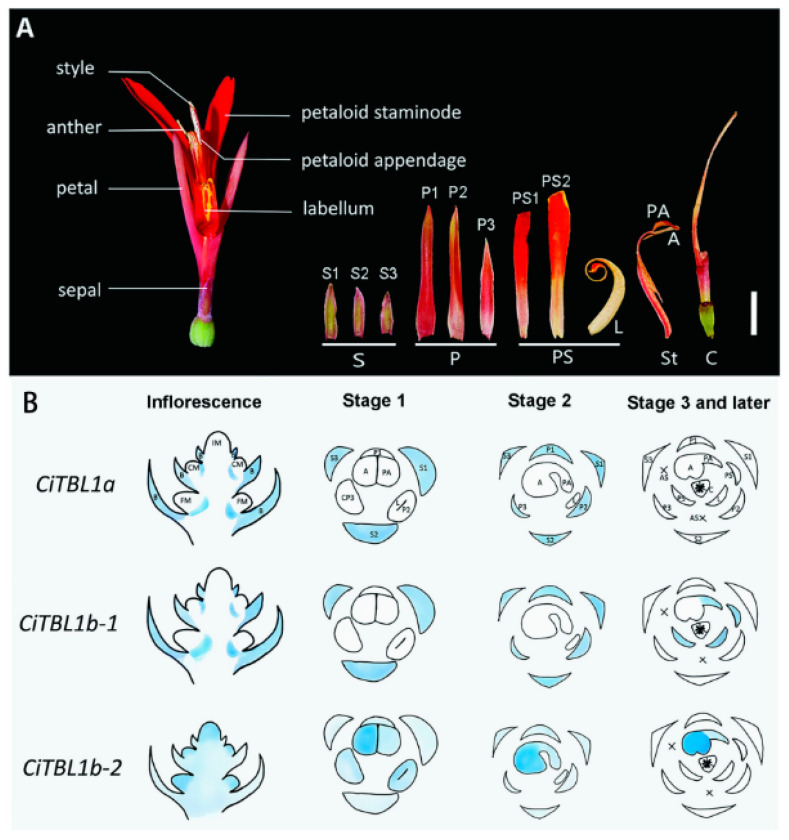
Morphology of *Canna indica* flowers and expression of *CiTBL* genes [142] (Yu et al., 2020). (**A**) Flower morphology. A: anthers; C: carpel; L: labellum; P: petals; PA: petaloid appendage; PS: petaloid staminodes; S: sepals; St: staminodes. (**B**) Expression of *CiTBL1a*, *CiTBL1b-1*, and *CiTBL1b-2* in young inflorescences and flowers at different developmental stages. Gene expression sites are indicated in blue, with the intensity of the coloration reflecting the expression level. AS: abortive staminodes; B: primary bracts; CM: meristem of monochasium; CP: common primordium of the petal and stamen; FM: floral meristem; IM: inflorescence meristem. Reprinted with permission from Ref. [142]. Copyright © 2023 Frontiers Media S.A.

*Canna indica* (*Cannaceae*) has noticeably asymmetrical flowers, in which the stamens develop into petal-like staminodes or are aborted (semi-fertile stamens) at an early developmental stage associated with three ECE clade homologs (*CiTBL1a*, *CiTBL1b-1*, and *CiTBL1b-2*) (Figure 8) [142]. The overexpression of *CiTBL* genes in *A. thaliana* resulted in dwarfism, the production of small petals and relatively few stamens, as well as mature flowers with altered symmetry, which provided evidence of the involvement of ECE clade homologs in the development of asymmetrical *C. indica* flowers.

### 7.4. CYC-like Genes of Ranunculales

*Ranunculaceae* underwent an evolutionary transition from actinomorphy to zygomorphy, with the accumulation of as many as four copies of the *CYC*-like gene *RanaCyL* in zygomorphic species [149,150]. The *RanaCyL* homologous genes are expressed early in flower bud development, and the expression duration varies with species and gene class. In actinomorphic species, at most one *RanaCyL* paralog is expressed late in flower development, whereas in zygomorphic species, all paralogs are expressed, constituting a species-specific recognition code for the perianth [149].

*Fumariaceae* and *Papaveraceae* show morphological diversity in flower symmetry and inflorescence structure, which may be related to the duplication and functional diversity of *CYC*-like genes [151]. Damerval et al. reported that the homologous genes of *CYC* in *Papaveraceae*, *PAPACYL1,* and *PAPACYL2*, which are ECE clade members, were expressed during the flower development of all three species studied and were specifically expressed in the outer petals of the two species with asymmetrical flowers [152].

The *CYC*-like *CYL* genes of the *Papaveraceae* species *Eschscholzia california* and *Cysticapnos vesicaria* have highly diverse expression patterns and functions [153]. The silencing of *EscaCYL1* enhances the control of bud branching, whereas *PapaCYL* genes promote germination and growth of stamens. In addition, *CyveCYL* genes are involved in the regulation of floral symmetry and perianth development of *Cysticapnos* by regulating B-class floral–organ identity genes to determine sepal and petal characteristics.

### 7.5. CYC-like Genes of other Families

An ECE clade gene, *CcCYC*, is not expressed in the radially symmetrical perianth of *Tradescantia pallida*, but is expressed asymmetrically in the bilaterally symmetrical perianth of *Commelina communis* and *Commelina dianthifolia* [138]. These observations were related to genes that were recruited in parallel through the independent evolution of flower bilateral symmetry in the early stage of floral development. The *Actinodium cunninghamii* (*Myrtaceae*) capitulum consists of a pseudanthium, with a ray flower that is not a single flower but a branch with a short bud that flowers occasionally; its proximal branch is also similar to the ray flower [139]. The changes in the expression of *CYC*-like genes in the pseudanthium modulated ray flower structures and branching patterns. This gene expression pattern is similar to that observed in the distantly related *Asteraceae* species, indicating that flowering plants seem to have recruited *CYC*-like genes at least twice in their evolutionary history for the development of heterotypic inflorescences.

Horn et al. showed that the ECE clade gene *CYCL* is present in basal angiosperms and *Magnoliaceae* species [140]. In *Aristolochia*, *CYCL* was involved in the differentiation of the perianth and the mushroom pseudo-structure, but did not participate in the process mediating the formation of zygomorphic flowers. Only when the TCP domain of the *Aristolochia CYCL* gene was replaced by the CYC2 domain could the functionally similar gene be obtained. The differentiation and evolution of the ECE lineage led to significant changes in the coding region and the *cis*-regulatory elements, which ultimately established *CYC2* as a key gene regulating floral zygomorphy in dicotyledons. Pabon-Mora et al. reported that ECE clade genes may also be involved in cell division in leaves, pistils, and ovules [154]. Specifically, *CYC*-like genes maintain differential expansion of the perianth by promoting cell division in the distal and ventral extremities during middle and late flower development in *Aristolochia fimbriata*.

Zhang and co-workers confirmed that the expression of the *CYC2*-like genes *CYC2A* and *CYC2B* was associated with the floral symmetry of *Malpighiaceae*, and that relaxation of their conserved expression and expansion to a wider floral area (including the dorsal stamen) were related to the development of dorsoventral heteranthery in *Hiptage benghalensis* and contributed to the elaborated androecium, which is essential for adaptation to the new pollination strategy [56,155]. Berger et al. compared the corolla shape of *Fedia graciliflora* expressing the wild-type or knocked-out *CYC2*-like gene *FgCYC2A* using canonical variable analysis, and observed that gene knockout resulted in significant changes in flower shape, which affected the position of the dorsal lobe relative to the lateral lobe and led to more radially symmetrical flowers [156].

Radially symmetric *Rhododendron taxifolium* and bilaterally symmetric *Rhododendron beyerinckianum* have four and five *CYC*-like genes from shared tandem duplications, respectively [28]. The *CYC*-like genes are expressed in the longer dorsal petals and stamens, and are highly expressed in the pistil of *R. beyerinckianum*, whereas in *R. taxifolium* the orthologs are either ubiquitously expressed, have been lost from the genome, or are weakly expressed [28]. As the main regulatory factor for the growth of differentiated organs in *Rhododendron*, *CYC*-like genes did not regulate the expression of *RAD*-like genes, which revealed a certain deviation from the typical floral symmetry-related gene regulatory network of asterids [28].

Three *CYC*-like genes (*CamCYC1*, *CamCYC2*, and *CamCYC3*) in *Campanulaceae* have undergone dynamic changes in replication and loss, including the first instance of the loss of *CamCYC2* in a bilaterally symmetrical group [27]. The *CamCYC1* gene was included in duplication events in the radially symmetrical *Campanuloideae* species, whereas *CamCYC2* was duplicated but *CamCYC3* was lost at an early stage of divergence, in the bilaterally symmetrical and inverted *Lobelioideae* species [27]. In addition, the bilaterally symmetrical and non-inverted *Cyphioideae* species lost *CamCYC2*, but replicated *CamCYC3* [27]. The late expression of *CamCYC2* along the dorsoventral axis of the inverted flower was confirmed, and was not regulated by external factors, such as gravity [27].

## 8. Outlook

Researchers have conducted systematic and detailed studies on the *CYC*-like genes of many angiosperm families, such as *Fabaceae*, *Asteraceae*, *Scrophulariaceae*, *Gesneriaceae*, and *Orchidaceae*. However there are still many issues regarding the function and evolution of *CYC*-like genes that require exploration in greater detail.

### 8.1. Conduct Systematic Functional and Evolutionary Research, Especially Regarding CYC1 and CYC3 Clade Members

The *CYC*-like genes have extensive and important roles affecting plant development [26,29,30]. The current relevant research has mainly focused on the *CYC2* clade, which is primarily associated with the regulation of floral symmetry, with less research conducted on the *CYC1* and *CYC3* clades [53,54,55,56,57,112]. Therefore, the functions and evolution of *CYC1* and *CYC3* genes should be investigated, to expand our understanding of the contributions of *CYC*-like genes to the growth and development of angiosperms.

### 8.2. Functionally Characterize the CYC-like Genes in More Plant Groups

Through developmental biology, genetics, and evolutionary genetics, scientists revealed that the ancestors of *CYC* in core eudicots were expressed in the dorsal flower organs, thus affecting floral symmetry [19,26,112,157]. The *CYC* genes are expressed in the ventral floral organs of several monocot groups (*Zingiberaceae, Alstroemeriaceae*, and *Commelinaceae*) [138,141,158]. In *Alstroemeriaceae*, Hoshino et al. observed that the *CYC*-like genes *AaTCP1*, *AmTCP1*, *ApTCP1*, and *ApTCP2*, which belong to the ECE clade, are involved in the development of floral asymmetry and the identity of ventral floral organs in *Alstroemeria aurea*, *Alstroemeria magenta*, and *Alstroemeria pelegrina* with bilaterally symmetrical flowers [158]. In addition, *AaTCP1* transcripts were specifically accumulated in flower buds and located at the paraxial perianth base of *A. aurea*. These results reflect the complexity of the *CYC* expression pattern in angiosperms. Additional research on these genes and their regulatory effects on floral symmetry will require the inclusion of more plant groups.

### 8.3. Investigate the Regulatory Elements Upstream of CYC-like Genes

Increasing numbers of studies have isolated and analyzed the phylogenetic relationships, expression patterns, and functions of *CYC*-like homologs in different angiosperm groups, but there has been minimal research on the upstream regulatory elements. Yang and co-workers determined that the bilateral symmetry of the flowers in *Gesneriaceae* may have involved the evolution of an automatic regulatory loop for the *CYC*-like gene [119,120]. In the *double-flowered* (*dbl*) sunflower mutant, *HaCYC2c* inserted into the promoter region is usually expressed specifically in wild-type ray florets, but not throughout the capitulum, possibly resulting in the inability to observe radially symmetrical flowers [79]. CmWUS can bind to the *cis*-acting element TAAT in the *CmCYC3a* promoter in yeast, and activate the expression of resistance genes, while also regulating floral symmetry and flower organ development together with ECE TFs in chrysanthemum [89]. The chrysanthemum TF CmCYC2c can bind to the *cis*-acting element of *CmCYC2f* to activate its expression, but it can also form heterodimers with CmCYC2c-2, CmCYC2d, and CmCYC2e, which may participate in the regulation of floral organ symmetry [88]. The spatiotemporal expression patterns and functions of *CYC*-like genes in different flower organs in different taxonomic groups are diverse, which may be related to changes in the upstream regulatory elements, ultimately resulting in a variety of angiosperm flower types. Therefore, the regulatory elements upstream of *CYC*-like genes must be studied, which will help to clarify the evolution and functional differentiation of these genes in angiosperms.

### 8.4. Study the Phylogenetic Relationships and Expression of CYC-like Genes with New Techniques and Methods

In addition to traditional methods for verifying gene functions, methylation analyses and other technical methods should be used to study the apparent modifications to *CYC*-like genes. Zhang et al. used qRT-PCR and bisulfite sequencing PCR techniques to determine the expression patterns and DNA methylation patterns of *CYC2*-like genes in two types of chrysanthemum florets, thereby providing new epigenetic-related insights into the formation of the capitulum in *Asteraceae* [98]. Sun et al. confirmed that *CYC2c* is the main factor influencing the *Gaillardia* ray–floret phenotype by applying RNA resequencing technology as well as qRT-PCR and gene-silencing methods [73]. In future studies, additional new technologies and methods including comparative genomics can be used to elucidate the role of *CYC*-like genes in angiosperm floral development, which will lead to new ideas for future research on the evolution and development of angiosperms.

## 9. Method

We randomly selected *CYC*-like genes from the published literature to construct a phylogenetic tree (Figure 1) using MEGA 11 software. The nucleotide sequences of the homologous *CYC* genes were downloaded from NCBI GenBank (https://www.ncbi.nlm.nih.gov/) (accessed on 9 October 2022) and aligned with ClustalW. The phylogenetic tree was constructed with MEGA 11 software using the maximum likelihood method [159]. The accession numbers of the sequence data used to construct the phylogenetic tree are present in the Supplemental Table S1. To assess support for the topology of the tree, a bootstrap analysis with 1000 replications was performed. In the phylogenetic tree, the CYC1, CYC2, and CYC3 clades are labeled with reference to the literature, whereas other *CYC*-like genes do not have an explicit classification at present [21,37,74,75,76].

## Data Availability

No confidential/unpublished data have been used in this article.

## Supplementary Materials

The following supporting information can be downloaded at: https://www.mdpi.com/article/10.3390/cimb45030131/s1, Supplementary Table S1. The accession numbers of the genes used to construct the phylogenetic tree.
