# Supplementary material for: Advances in Research on the Regulation of Floral Development by CYC-like Genes
Source: Curr Issues Mol Biol. 2023 Mar 2;45(3):2035–59. doi: 10.3390/cimb45030131 (PMC10047570; doi:10.3390/cimb45030131)
Supplement: Supplementary file 1 [file cimb-45-00131-s001.zip › cimb-2191178-supplementary.pdf]

Supplementary Table S1. The accession numbers of the genes used to construct the phylogenetic tree.

GENE NAME	ACCESSION NUMBER	SPECIES
AmDICH	AAF12817.1	*Antirrhinum majus*
AmCYC1	Q9SBV9.1	*Antirrhinum majus*
AmCYC2	O49250.1	*Antirrhinum majus*
CmCYC2a	KU595430.1	*Chrysanthemum morifolium*
CmCYC2b	KU595431.1	*Chrysanthemum morifolium*
CmCYC2d	KU595426.1	*Chrysanthemum morifolium*
CmCYC2e	KU595427.1	*Chrysanthemum morifolium*
CmCYC2f	KU595429.1	*Chrysanthemum morifolium*
CvCYC	AF512601.1	*Chaenorrhinum villosum*
GhCYC1a	AEX07367.1	*Gerbera hybrid*
GhCYC1b	ACC54346.1	*Gerbera hybrid*
GhCYC2a	ACC54347.1	*Gerbera hybrid*
GhCYC2b	ACC54348.1	*Gerbera hybrid*
GhCYC2c	ACC54349.1	*Gerbera hybrid*
GhCYC2d	AEX07362.1	*Gerbera hybrid*
GhCYC2e	AEX07364.1	*Gerbera hybrid*
GhCYC2f	AEX07366.1	*Gerbera hybrid*
GhCYC3a	AEX07365.1	*Gerbera hybrid*
GhCYC3b	AEX07363.1	*Gerbera hybrid*
HaCYC1a	ABV26440.1	*Helianthus annuus*
HaCYC1b	ABV26441.1	*Helianthus annuus*
HaCYC2a	ABV26442.1	*Helianthus annuus*
HaCYC2b	ABV26443.1	*Helianthus annuus*
HaCYC2c	ABV26444.1	*Helianthus annuus*
HaCYC2d	ABV26445.1	*Helianthus annuus*
HaCYC2e	ABV26446.1	*Helianthus annuus*
HaCYC2f	XP_022034966.1	*Helianthus annuus*
HaCYC3a	ABV26447.1	*Helianthus annuus*
HaCYC3b	ABV26448.1	*Helianthus annuus*
HaCYC3c	ABV26449.1	*Helianthus annuus*
LjCYC1	ABB36471.1	*Lotus japonicus*
LjCYC2	ABB36472.1	*Lotus japonicus*
LjCYC3	ABB36473.1	*Lotus japonicus*
MoCYC	AF512600.1	*Misopates orontium*
ObCYC1	ACY82350.1	*Oreocharis benthamii*
ObCYC2	ACY82349.1	*Oreocharis benthamii*
OdCYC1C	ACY82351.1	*Oreocharis dinghushanensis*
OdCYC1D	ACY82352.1	*Oreocharis dinghushanensis*
PcCYC1	AAR99880.1	*Paulownia tomentosa*
PeCYC2	KT258892.1	*Phalaenopsis equestris*
PeCYC3	KT258892.1	*Phalaenopsis equestris*
PgCYC1C	ALE27657.1	*Petrocosmea glabristoma*

PgCYC1D	ALE27658.1	*Petrocosmea glabristoma*
PhCYC1C	AFN66834.1	*Primulina heterotricha*
PhCYC1D	AFN66835.1	*Primulina heterotricha*
PhTCP2	AKQ19160.1	*Primulina heterotricha*
PsCYC1C	ALE27664.1	*Petrocosmea sinensis*
PsCYC1D	ALE27665.1	*Petrocosmea sinensis*
PsCYC2	ADX59554.1	*Petrocosmea sinensis*
RgCYC	AAX39411.1	*Rehmannia glutinosa*
SaRAY2	JQ351911.1	*Senecio aethnensis*
SacRAY2	JQ351929.1	*Senecio aethnensis*
ScRAY2	JQ351921.1	*Senecio chrysanthemifolius*
SiCYC1A	AAZ15874.1	*Streptocarpus ionanthus*
SiCYC1B	AAZ15876.1	*Streptocarpus ionanthus*
SoCYC2e	MG593440.1	*Sinosenecio oldhamianus*
SsCYC	URQ29610.1	*Senecio squalidus*
SscCYC2e	MG593448.1	*Senecio squalidus*
SsRAY2	FJ356704.1	*Senecio squalidus*
SvCYC1	ACJ71723.1	*Senecio vulgaris*
SvCYC2	ACJ71727.1	*Senecio vulgaris*
SvRAY1	ACJ71723.1	*Senecio vulgaris*
SvRAY2	ACJ71727.1	*Senecio vulgaris*
VrCYC3	T0011489.1	*Vigna radiata*
